# Modified look-locker inversion recovery T1 mapping indices: assessment of accuracy and reproducibility between magnetic resonance scanners

**DOI:** 10.1186/1532-429X-15-64

**Published:** 2013-07-26

**Authors:** Fabio S Raman, Nadine Kawel-Boehm, Neville Gai, Melanie Freed, Jing Han, Chia-Ying Liu, Joao AC Lima, David A Bluemke, Songtao Liu

**Affiliations:** 1 Radiology and Imaging Sciences, National Institutes of Health Clinical Center, 10 Center Drive, MSC-1182, Bethesda, MD 20892-1182, USA; 2 Clinic of Radiology and Nuclear Medicine, University of Basel Hospital, Basel, Switzerland; 3 New York University School of Medicine, New York, NY, USA; 4 U.S. Food and Drug Administration, Rockville, MD, USA; 5 Division of Cardiology, Johns Hopkins University School of Medicine, Baltimore, MD, USA; 6 Molecular Biomedical Imaging Laboratory, National Institute of Biomedical Imaging and Bioengineering, Bethesda, MD, USA

**Keywords:** T1 mapping, Partition coefficient (λ), Extracellular volume fraction (ECV), Diffuse myocardial fibrosis, Modified look-locker with inversion recovery (MOLLI)

## Abstract

**Background:**

Cardiovascular magnetic resonance (CMR) T1 mapping indices, such as T1 time and partition coefficient (λ), have shown potential to assess diffuse myocardial fibrosis. The purpose of this study was to investigate how scanner and field strength variation affect the accuracy and precision/reproducibility of T1 mapping indices.

**Methods:**

CMR studies were performed on two 1.5T and three 3T scanners. Eight phantoms were made to mimic the T1/T2 of pre- and post-contrast myocardium and blood at 1.5T and 3T. T1 mapping using MOLLI was performed with simulated heart rate of 40-100 bpm. Inversion recovery spin echo (IR-SE) was the reference standard for T1 determination. Accuracy was defined as the percent error between MOLLI and IR-SE, and scan/re-scan reproducibility was defined as the relative percent mean difference between repeat MOLLI scans. Partition coefficient was estimated by ΔR1myocardium phantom/ΔR1blood phantom. Generalized linear mixed model was used to compare the accuracy and precision/reproducibility of T1 and λ across field strength, scanners, and protocols.

**Results:**

Field strength significantly affected MOLLI T1 accuracy (6.3% error for 1.5T vs. 10.8% error for 3T, p<0.001) but not λ accuracy (8.8% error for 1.5T vs. 8.0% error for 3T, p=0.11). Partition coefficients of MOLLI were not different between two 1.5T scanners (47.2% vs. 47.9%, p=0.13), and showed only slight variation across three 3T scanners (49.2% vs. 49.8% vs. 49.9%, p=0.016). Partition coefficient also had significantly lower percent error for precision (better scan/re-scan reproducibility) than measurement of individual T1 values (3.6% for λ vs. 4.3%-4.8% for T1 values, approximately, for pre/post blood and myocardium values).

**Conclusion:**

Based on phantom studies, T1 errors using MOLLI ranged from 6-14% across various MR scanners while errors for partition coefficient were less (6-10%). Compared with absolute T1 times, partition coefficient showed less variability across platforms and field strengths as well as higher precision.

## Background

Diffuse myocardial fibrosis is a common feature of a broad variety of cardiovascular diseases. Cardiovascular magnetic resonance (CMR) T1 mapping indices, including T1 time, partition coefficient (λ), and extracellular volume fraction (ECV) measurements, have all been used to estimate the expansion of myocardial interstitial space thought to be associated with myocardial fibrosis [1-3]. T1 mapping indices are altered in a variety of cardiomyopathies, including chronic aortic regurgitation [4], heart failure [5], aortic stenosis [6], and adult congenital heart disease [7]. Compared with absolute T1 time, partition coefficient and ECV are relatively stable *in-vivo* in mild interstitial expansion conditions approximately 10 min after intravenous gadolinium based contrast agent (GBCA) administration [8,9]. They are less sensitive to magnetic field strength and are similar between different GBCAs [10,11]. ECV, in particular, has been linked to disease-specific changes and correlate with CMR parameters of disease severity [12].

MOLLI determination of T1 offers tighter limits of agreement between repeated measurements than traditional T1 mapping techniques, such as Look-Locker or multibreath-hold FLASH inversion recovery sequences [13,14]. T1 determination by MOLLI can be influenced by multiple scanner dependent factors, such as field strength, gradient systems, coil configuration, pulse sequence design, artifacts related to field inhomogeneity and eddy currents. Those factors are directly related to the scanner’s performance, and therefore, are usually site/scanner-dependent and time-variant. However, to the best of our knowledge, the variability of MOLLI T1 measurement has not been well established between different scanners and static magnetic field strengths. Understanding MOLLI T1 variability is highly relevant to multi-center studies as well as follow-up of an individual patient using a different MR scanner than at baseline.

The purpose of the study was to determine the variation in T1 and partition coefficient between MR scanners using MOLLI in comparison to a set of reference T1 values. For this evaluation, we measured MOLLI T1 values in comparison to standard of reference inversion-recovery spin-echo (IR-SE) phantom vials that mimicked the T1 and T2 values of pre- and post-contrast myocardium and blood pool measurements at 1.5 and 3T.

## Methods

### Phantom

Eight phantoms total, or a set of four phantoms for each 1.5T and 3T, were made from a NiCl_2_-DTPA/agarose solution to evaluate a range of pre- and post-contrast myocardium and blood pool T1 values [15]. The concentrations of reagents were adjusted to mimic the T1 and T2 values of the normal myocardium and blood pool [9,16,17].

### CMR parameters

A list of the scanners with vendor, field strength, and coil information are shown in Table 1. Two different MOLLI acquisitions were performed on phantoms on all of the scanners with a simulated heart rate ranging from 40–100 beats per minute. The standard 17 heart beat MOLLI protocol has three inversion blocks; three images are acquired after each of the first two inversion pulses (3a), followed by a pause of three heart beats (3p), then five images are acquired after a third inversion pulse (5a). The shortened 11 heart beat MOLLI protocol has two inversion blocks; three images are acquired after the first inversion pulse (3a), followed by a pause of three heart beats (3p), then five images are acquired after a second inversion pulse (5a). Parallel imaging with a phase reduction factor of two was used. Localized shim and frequency scout was performed before MOLLI acquisition to improve static magnetic field homogeneity [18]. The full CMR parameters are listed in Table 2. Due to inherent differences in scanner performance and software, there are minor differences in sequence parameters as shown, but these variations were kept to a minimum and would not be expected to affect T1 estimation.

**Table 1:** CMR scanners used for experiments

**Scanner**	**Vendor**	**Model**	**Field strength**	**Coil**	**Maximum gradient strength (mT/m)**	**Slew rate (mT/m/s)**
**1**	Siemens Medical Solutions	Avanto	1.5T	12Ch	45	200
**2**	Philips Healthcare	Achieva	1.5T	16Ch	33	122
**3**	Siemens Medical Solutions	Verio	3.0T	32Ch	45	200
**4**	Philips Healthcare	Achieva	3.0T	16Ch	40	200
**5**	Philips Healthcare	Achieva	3.0T	16Ch	40	200

Note: Table displays the different scanners used in the study with their specifications.

**Table 2:** MOLLI scan parameters

**Parameter**	**1.5T**		**3.0T**	
	**Siemens**	**Philips**	**Siemens**	**Philips**
**TR (ms)**	2.4	2.3	2.4	2.4
**TE (ms)**	1.0	1.0	1.0	0.95
**Flip Angle (degrees)**	35			
**Minimum TI (ms)**	125	120	95	135
**TI Increment (ms)**	80			
**Pixel Bandwidth (Hz/pixel)**	1002	1042	1002	1036
**Field of View (mm)**	285 × 360			
**Image Matrix**	192 × 124			
**Slice Thickness (mm)**	8			
**Encoding Order**	Linear			
**Readout**	Non-segmented steady state free precession			

Note: Table displays the CMR parameters used in the study across the different field and vendors.

Reference T1 times on each scanner were determined using standard inversion recovery prepared spin echo sequences at 10 different TIs from 22 to 5,000 ms (TR=10,000 ms, TE=9 ms), with the same FOV, matrix size, and slice thickness as the MOLLI experiment. For precision/reproducibility measurements, the phantoms were taken out of the magnet after the MOLLI and IR-SE images were acquired. Phantoms were then repositioned into the magnet 10–15 minutes later and a second set of MOLLI images acquired using the exact same parameters. The total average delay between two MOLLI scans was 8 hours. Our method of testing the true reproducibility measurements by leaving a long time span between the scans and repositioning the phantom resembles those of clinical reproducibility studies [19,20], allowing these results to be more directly transferrable for *in-vivo* assessment.

### Image analysis

Both inversion recovery and MOLLI T1 maps were calculated using MRmap [21]. T1 time was calculated with a 3-parameter curve fitting using a Levenberg-Marquardt algorithm and additional T1* correction was applied for MOLLI data.

(1) $$y=A-B\exp\left(-\mathrm{TI}/T1\right)$$

(2) $$T1=T1*\left(\left(B/A\right)-1\right)$$

ImageJ was used to measure the T1 values for each of the vials. Partition coefficient (λ) was calculated according to the following formulae [8]:

(3) $${\Delta R1}_{\mathrm{myocardium}}=1/\left({T1}_{\mathrm{post}-\mathrm{myo}}\right)-1/\left({T1}_{\mathrm{pre}-\mathrm{myo}}\right)$$

(4) $${\Delta R1}_{\mathrm{blood}}=1/\left({T1}_{\mathrm{post}-\mathrm{blood}}\right)-1/\left({T1}_{\mathrm{pre}-\mathrm{blood}}\right)$$

(5) $$\lambda ={\mathrm{\Delta R}1}_{\mathrm{myocardium}}/{\mathrm{\Delta R}1}_{\mathrm{blood}}$$

### Accuracy assessment

Accuracy was assessed for T1 mapping indices with IR-SE as a reference. The percent error between MOLLI and IR-SE was calculated for both T1 values and partition coefficient. The error was defined in the following equations:

(6) $$T1\;\mathrm{error}\;\left(\%\right)=\frac{\left|\mathrm{MOLL}I_{T_{1}}-\mathrm{IRS}E_{T_{1}}\right|}{\mathrm{IRS}E_{T_{1}}}\times 100\%$$

(7) $$\mathrm{Partition}\;\mathrm{coefficient}\;\mathrm{error}\;\left(\%\right)=\frac{\left|\mathrm{MOLL}I_{\lambda }-\mathrm{IRS}E_{\lambda }\right|}{\mathrm{IRS}E_{\lambda }}\times 100\%$$

Smaller error numbers represent better accuracy.

### Precision assessment (test-retest reproducibility)

Precision (test-retest reproducibility) was assessed by measuring the agreement between independent MOLLI acquisitions. The relative mean percent difference between the two MOLLI scans was calculated for both T1 values and partition coefficient reproducibility. Smaller error numbers represent better precision.

(8) $$\mathrm{Relative}\;\mathrm{mean}\;\mathrm{difference}=\frac{\left|\mathrm{MOLL}I_{\mathrm{Scan}1}-\mathrm{MOLL}I_{\mathrm{Scan}2}\right|}{\mathrm{MOLL}I_{\mathrm{average}}}\times 100\%$$

### Statistical analysis

Statistical analyses were performed using SAS 9.1 (Cary, North Carolina, USA) and MedCalc 12.2 (MedCalc Software, Mariakerke, Belgium). Statistical significance was defined as *p*<0.05. In order to statistically compare the T1/partition coefficient accuracy and reproducibility across field strengths, scanners, and protocols, a general linear mixed model was used. The scanners, field strengths, and MOLLI protocols were included as a fixed effect. Results were reported as least squared means ± standard error. Bland-Altman plots were used to describe the difference between scan repetitions for both native T1 and partition coefficient values.

## Results

CMR studies were performed on two 1.5T (1 Philips, 1 Siemens) and three 3T (2 Philips, 1 Siemens) scanners. Four phantoms were analyzed per scan for two MOLLI protocols at seven different heart rates for five scanners with two repetitions, totaling 560 MOLLI T1 measurements. All scanners are operating within manufacturer’s specification.

### Comparison of T1 and partition coefficient

T1 values for both IR-SE and MOLLI are shown in Table 3. MOLLI T1 values were significantly different between scanners within the same field strength (p<0.0001 for 1.5T and 3T). MOLLI partition coefficients were not significantly different between 1.5T scanners (47.2% vs. 47.9%, p=0.13) as shown in Table 3. On the other hand, MOLLI partition coefficients were significantly different among 3T scanners (p=0.016). However, the differences among 3T scanners were very small (49.2% vs. 49.8% vs. 50.0%, Table 3) and probably wouldn’t have any clinical implication.

**Table 3:** IR-SE, MOLLI T1 and partition coefficient values

	**T1 (ms)**				**Partition coefficient (%)**
	**Pre-contrast vials**		**Post-contrast vials**		
	**“Myocardium”**	**“Blood Pool”**	**“Myocardium”**	**“Blood Pool”**	
**1**	**869** (773)	**1408** (1333)	**450** (416)	**316** (322)	**43.7** (47.2)
**2**	**892** (814)	**1434** (1369)	**468** (435)	**331** (337)	**43.7** (47.9)
**3**	**1528** (1332)	**1894** (1750)	**603** (575)	**367** (391)	**45.8** (49.2)
**4**	**1643** (1253)	**2041** (1693)	**649** (593)	**398** (423)	**46.0** (49.8)
**5**	**1537** (1272)	**1882** (1630)	**606** (583)	**370** (400)	**46.2** (50.0)
	**T2 (ms)**				
**1.5T**	40	290	53	179	
**3T**	51	273	49	166	

Note: IR-SE T1 and partition coefficient values are shown in bold. MOLLI T1 and partition coefficient values are displayed inside parenthesis. Data obtained from 4 vials that mimic the T1/T2 of pre- and post-contrast myocardium and blood pool from the 5 scanners. T2 values for both 1.5T and 3T are displayed at the bottom of the table.

### Accuracy assessment

**Figure 1:**
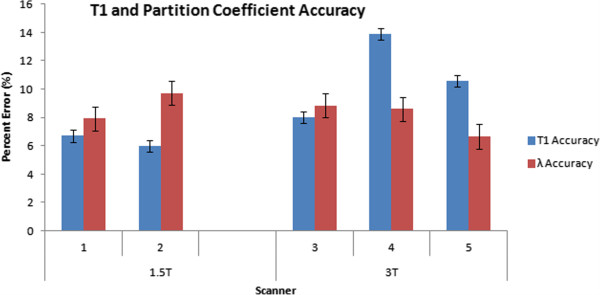
**MOLLI T1 and partition coefficient accuracy with different scanners.** Bar graph representation of accuracy for MOLLI T1 and partition coefficient for each of the 5 scanners. Accuracy is reported as the percentage difference between MOLLI and IR-SE. Data is presented as least square means ± standard error. Smaller numbers represent better accuracy.

The mean percent error of MOLLI T1 values are displayed in Figure 1 and comparison of field strengths and protocols are summarized in Table 4. The MOLLI T1 errors were 6-14% across various MR scanners, while the errors for partition coefficient were less (6-10%) between MR scanners (Figure 1). There was no statistical significance in MOLLI T1 accuracy between the 17HB protocol and 11HB protocol (8.3% vs. 8.8%, p=0.18), so these values were combined for subsequent analyses. Mean percentage T1 errors were 6.7 ± 0.5%, 6.0 ± 0.4%, 8.0 ± 0.4%, 13.9 ± 0.4%, and 10.6 ± 0.4% for scanners 1 thru 5, respectively (least square means ± standard error).

**Table 4:** MOLLI T1 and partition coefficient accuracy and reproducibility across field strengths and protocols

	**Field strength**			**Protocol**		
	**1.5T**	**3T**	**p-value**	**17HB**	**11HB**	**p-value**
**Accuracy (% error)**						
**T1 accuracy**	6.3 ± 0.3	10.8 ± 0.2	<0.0001	8.3 ± 0.3	8.8 ± 0.3	0.18
**Partition coefficient accuracy**	8.8 ± 0.6	8.0 ± 0.5	0.11	8.3 ± 0.6	8.5 ± 0.6	0.57
**Reproducibility (% mean difference)**						
**T1 reproducibility**	5.3 ± 0.3	3.9 ± 0.2	<0.0001	4.5 ± 0.2	4.7 ± 0.2	0.43
**Partition coefficient reproducibility**	3.8 ± 0.3	2.8 ± 0.2	0.02	2.7 ± 0.3	4.0 ± 0.3	0.003

Note: The accuracy and reproducibility of MOLLI on field strength and protocol are reported. Accuracy is reported as the percentage difference between MOLLI and IR-SE. Scan/rescan reproducibility is reported as the relative percent mean difference between two MOLLI scans. Data is presented as least square means ± standard error. Smaller numbers represent better accuracy or reproducibility.

**Figure 2:**
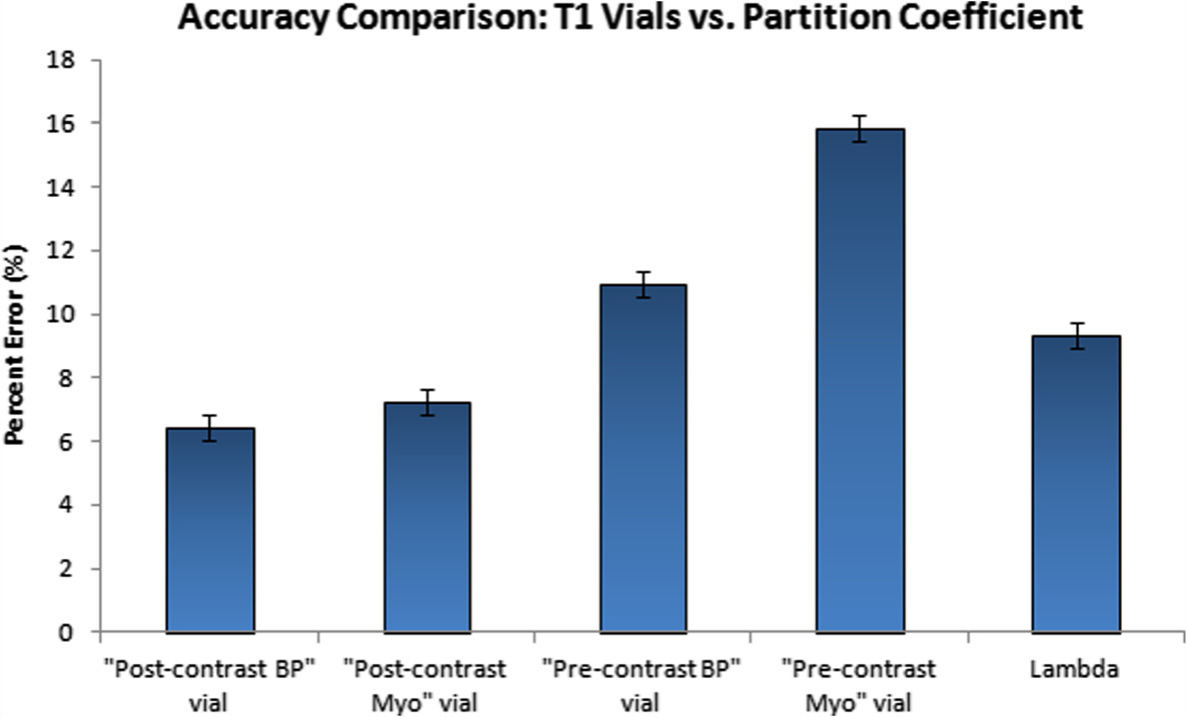
**Accuracy measurements for different vials.** Bar graph representation comparing MOLLI T1 accuracy measurements across four different vials. Accuracy is reported as the percentage difference between MOLLI and IR-SE. Data is presented as least square means ± standard error. Smaller numbers represent better accuracy. The two post-contrast vials with lower T1 times had better accuracy than the two pre-contrast vials with longer T1 values. The accuracy of partition coefficient is in the middle of pre and post-contrast vials.

1.5T scanners had smaller percent error, and hence better T1 accuracy, than 3T scanners (6.3 ± 0.3% for 1.5T vs. 10.8 ± 0.2% for 3T, p<0.0001). T1 accuracy for individual vials was also compared to look at subtleties as shown in Figure 2. The two post-contrast vials with lower T1 times had better accuracy (6.4 ± 0.4% and 7.2 ± 0.4%) than the two pre-contrast vials with longer T1 values (11.0 ± 0.4% and 15.9 ± 0.4%). The partition coefficient accuracy lies in the middle of pre and post-contrast vials (9.4 ± 0.3%).

The reference partition coefficient values estimated from IR-SE were 43.7% for 1.5T and 46.0% for 3T, corresponding to ECV values of 26.2% and 27.6%, respectively, if an average hematocrit of 0.4 is assumed. These values were similar to normal in-vivo ECV values from literature, 25.4% at 1.5T [17] and 26.7% at 3T [8]. Partition coefficient accuracy varied significantly between different scanners (p<0.0001). Average percentage partition coefficient errors were 7.9 ± 1.0%, 9.7 ± 0.9%, 8.9 ± 0.9%, 8.6 ± 0.9%, and 6.7 ± 0.9% for scanners 1 thru 5, respectively (least square means ± standard error, Figure 1). Field strength did not significantly affect partition coefficient accuracy (8.8 ± 0.6% for 1.5T vs. 8.0 ± 0.5% for 3T, p=0.109). In addition, different MOLLI protocols did not significantly affect partition coefficient accuracy (8.3 ± 0.6% for 17HB vs. 8.5 ± 0.6% for 11HB, p=0.574). Both T1 (p<0.001) and partition coefficient (p=0.001) accuracy vary significantly across heart rate.

### Precision/reproducibility assessment

**Figure 3:**
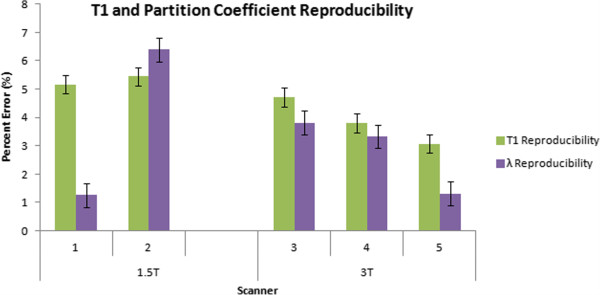
**MOLLI T1 and partition coefficient reproducibility with different scanners.** Bar graph representation of reproducibility measurements for both T1 and partition coefficient for each of the 5 scanners as well as field strength, and protocols. Scan/rescan reproducibility is reported as the relative percent mean difference between two MOLLI scans. Data is presented as least square means ± standard error. Smaller numbers represent better reproducibility.

T1 reproducibility varied significantly between different scanners (p=0.007). Average T1 differences were 5.2 ± 0.4%, 5.5 ± 0.3%, 4.7 ± 0.3%, 3.8 ± 0.3%, and 3.1 ± 0.3% for scanners 1 thru 5, respectively (least square means ± standard error, Figure 3). T1 reproducibility did not vary significantly across heart rates (p=0.375). However, field strength did significantly affect T1 precision with 3T showing smaller percent error, or better scan/re-scan reproducibility, than 1.5T (5.3 ± 0.3% for 1.5T vs. 3.9 ± 0.2% for 3T, p<0.0001, Table 4).

Partition coefficient precision/reproducibility varied significantly between different scanners (p<0.0001). Average partition coefficient differences were 1.3 ± 0.6%, 6.4 ± 0.4%, 3.8 ± 0.4%, 3.3 ± 0.4%, and 1.3 ± 0.4% for scanners 1 thru 5, respectively (least square means ± standard error, Figure 3). Partition coefficient precision did not vary significantly across heart rates (p=0.24). However, field strength did significantly affect partition coefficient precision with 3T showing higher precision than 1.5T (3.8 ± 0.3% for 1.5T vs. 2.8 ± 0.2% for 3T, p=0.021, Table 4).

**Figure 4:**
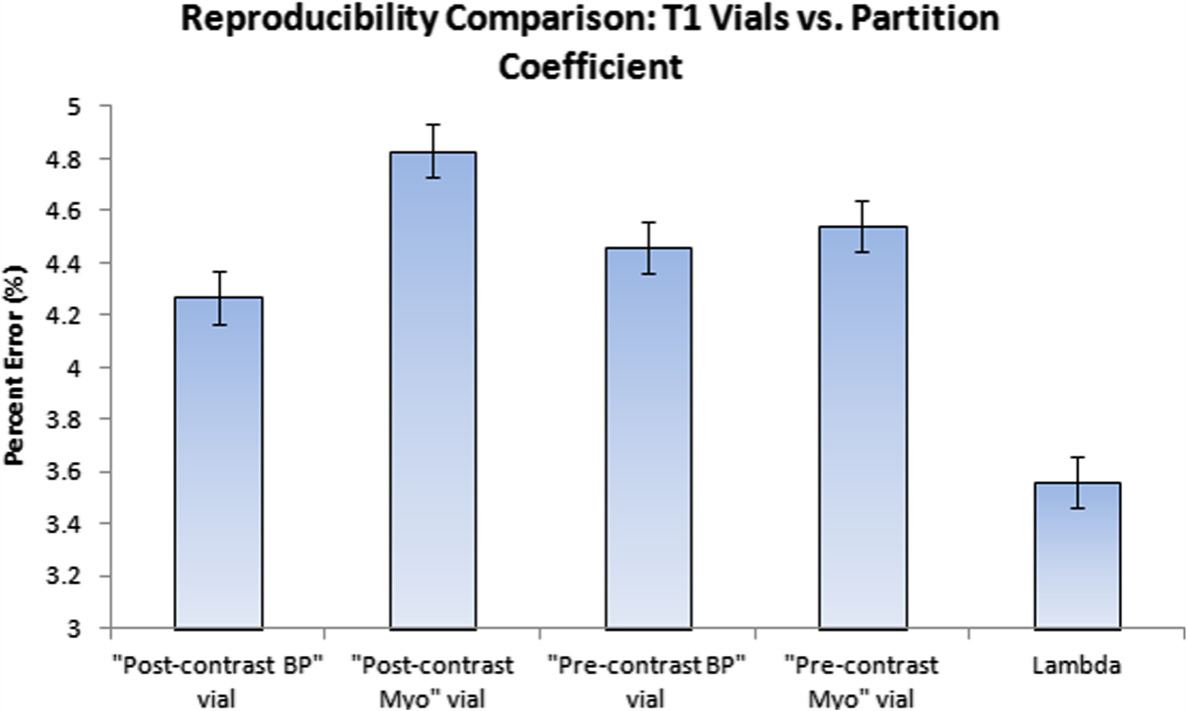
**Reproducibility measurements for individual vials.** Bar graph representation comparing MOLLI T1 reproducibility measurements across four different vials. Scan/rescan reproducibility is reported as the relative percent mean difference between two MOLLI scans. Smaller numbers represent better reproducibility. Data is presented as least square means ± standard error. Partition coefficient had significantly better scan/re-scan reproducibility than three of the four T1 vials with “post-contrast blood” vial being the only exception.

**Figure 5:**
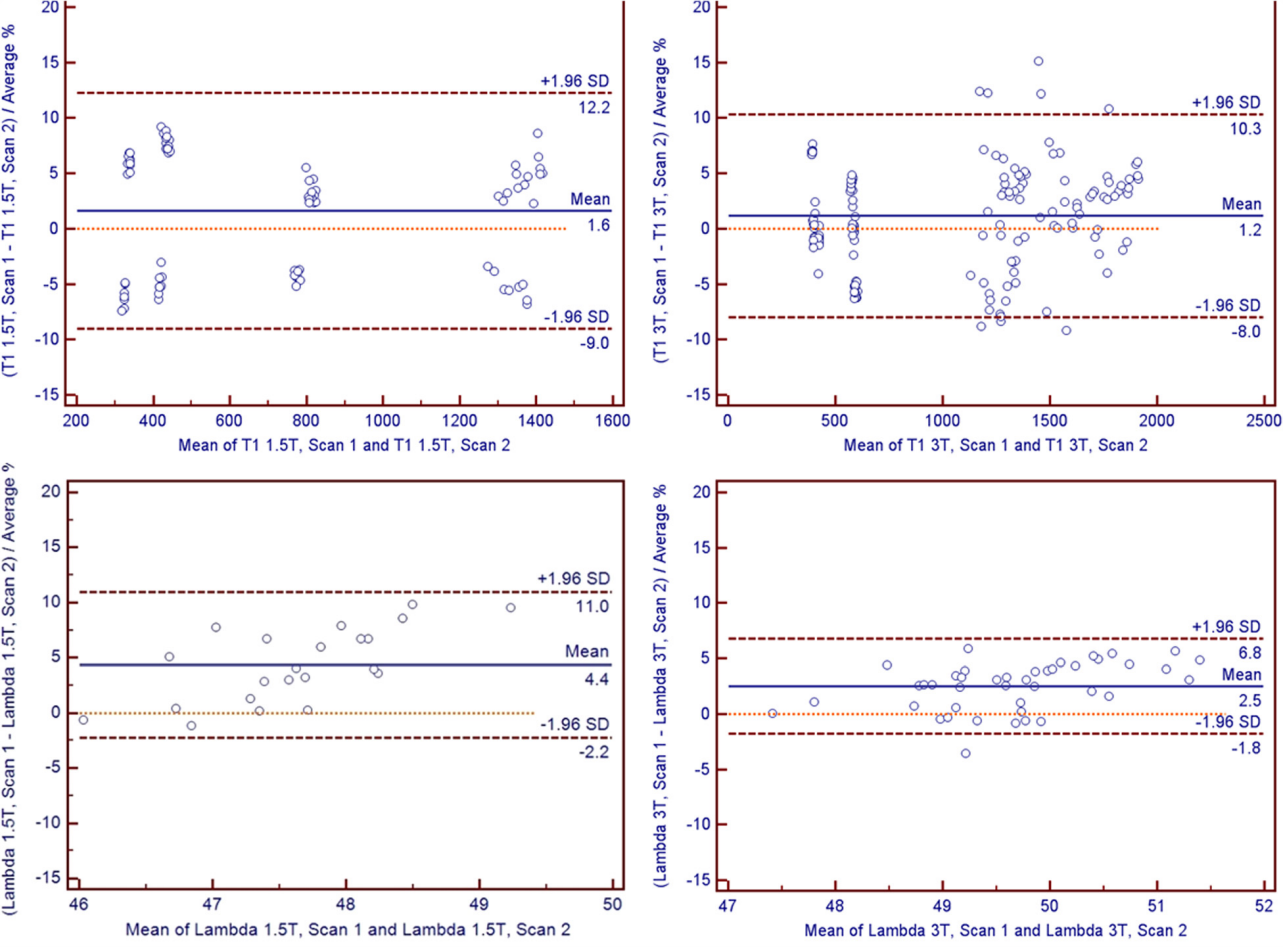
**Reproducibility: Bland-Altman.** Bland-Altman plots show partition coefficient and T1 reproducibility. Mean (bias) and 95% confidence interval limits of agreement are displayed. All values are presented as percentage differences.

When compared individually, partition coefficient had significantly better precision (scan/re-scan reproducibility) than three of the four T1 vials with the “post-contrast blood” vial being the only exception (p=.096 for “post-contrast blood” vial vs. p<0.05 for all other vials). There was no statistical difference of precision when comparing individual T1 vials with each other. Precision was 3.6% for partition coefficient compared to 4.3%, 4.8%, 4.5%, and 4.5% for the four different T1 vials (Figure 4). Bland-Altman analysis in Figure 5 revealed tighter 95% confidence interval (CI) limits of agreement for partition coefficient over MOLLI T1 for both 1.5T and 3T (1.5T: -9.0% to 12.2% for T1 vs. -11.0% to 2.2% for partition coefficient; 3T: -8.0% to 10.3% for T1 vs. -1.8% to 6.8% for partition coefficient).

## Discussion

CMR T1 mapping indices are novel non-invasive imaging biomarkers for myocardial extracellular space measurement [22-24]. There is increasing evidence supporting the clinical utility of T1 mapping quantification as well as of the serial assessment of such values. Multi-center studies using different MR platforms are ultimately required to assess the robustness of T1 measurements as a biomarker. Our major findings are: 1) T1 values by both IR-SE and MOLLI were different across scanners in the same field strength; 2) there was no difference between partition coefficients of 1.5T scanners (47.2% vs. 47.9%, p=0.13) and only slight differences across 3T scanners (49.2% vs. 49.8% vs. 49.9%, p=0.016); 3) partition coefficient had less variability in accuracy across platforms and field strength (8.8% error for 1.5T vs. 8.0% error for 3T, p=0.11) than MOLLI T1 values (6.3% error for 1.5T vs. 10.8% error for 3T, p<0.001); 4) partition coefficient had higher scan/re-scan reproducibility than MOLLI T1 values. In summary, the partition coefficient may be more robust than MOLLI T1 values when comparing between different scanners and field strengths. It is important to note that although only partition coefficient, and not ECV, was evaluated due to lack of hematocrit data, these results can be directly translated to ECV for clinical relevance as the only difference between partition coefficient and ECV is hematocrit correction.

### Accuracy

Accuracy was defined as the closeness of a MOLLI measurement to a reference IR-SE value, which was represented by percent error between MOLLI and IR-SE in this study. High T1/ECV accuracy will be particularly important for low to moderate extracellular matrix expansion diseases such as aging [25], diabetes [26], and hypertension; compared to diseases with substantial expansion like cardiac amyloidosis [27]. Different accuracy levels due to system differences will increase the inter-site variability and decrease the power of statistical comparison. Our data shows that IR-SE T1 values were different across scanners within the same field strength, validating our method of using scanner-specific IR-SE values to calculate accuracies. 17HB or 11HB protocol did not significantly affect either MOLLI T1 or partition coefficient values (p=0.177 for T1 vs. p=0.574 for partition coefficient). These results are consistent with previous reports on 1.5 and 3T scanners [8,28]. Unlike MOLLI T1, partition coefficient accuracy was not dependent on field strength (p<0.0001 for T1 vs. p=0.109 for partition coefficient). Within the same field strength, MOLLI T1 values were significantly different across scanners (p<0.0001 for both 1.5T and 3T). However, partition coefficient had no significant difference among 1.5T scanners (p=0.13) and only had a very small difference among 3T scanners (49.2% vs. 49.8% vs. 50.0%, p=0.016).

Newer T1 mapping techniques, such as shortened modified look-locker inversion recovery (shMOLLI) [29] and saturation recovery sequences [30,31], which are insensitive to heart rate variation were not available to us at the time of the study. ShMOLLI reduces acquisition time by using sequential inversion recovery measurements in a 5a+1a+1a sampling scheme each separated by only one R-R interval, which does not allow for full recovery of the magnetization for the three inversion pulses. Post-processing of images helps to correct for this problem by excluding data samples that fall outside accurate threshold. Saturation recovery sequences has demonstrated to have less T2 dependence and good agreement with IR-SE, as well as improved acquisition efficiency. As pre- and post-contrast T1 values are inherently different, an adaptive protocol (conditional sampling scheme) that optimizes for specific T1 ranges should improve accuracy. Preliminary data has shown subtle improvements of one such adaptive scheme over the standard MOLLI [32] with less variation at higher heart rates for 1.5T [9,33]. In addition, latest MOLLI sequence has offered the flexibility of a fixed time pause in seconds instead of the traditional heartbeats between inversion recovery blocks. This improvement is important for proper magnetization recovery in subjects with fast heart rates, especially at 3T where the pre contrast T1 value is longer.

It is well know that considerable spatial variation in transmit field (B1) exists even at 1.5T and adiabatic pulses are used to reduce the sensitivity to B1 field inhomogeneity. Kellman and et al. demonstrated that a shorter tangent/hyperbolic pulse outperforms the traditional hyperbolic secant pulse with an improved inversion factor of 0.96 [34]. This improved inversion efficiency translates directly into higher MOLLI T1 accuracy and meet the specific absorption rate requirement of both 1.5T and 3T. Future research should explore new T1 acquisition techniques to more accurately assess myocardial and blood pool T1.

### Precision/reproducibility

Reproducibility or precision is defined as the degree of closeness to which repeated measurements show similar results, which was measured by relative mean percent error between MOLLI scan repetitions. High reproducibility/precision leads to greater reliability of observed changes for a given parameter and significant reduction in sample size needed, as sample size varies with the square of the reproducibility.

Liu et al. reported good ECV and partition coefficient reproducibility for a single center; however, a lack of ECV and partition coefficient reproducibility/precision data exists for multicenter studies [20]. In this study, we demonstrated that although both MOLLI T1 and partition coefficient reproducibility were different across scanners and affected by field strength, they were not affected by heart rate (p=0.432 for T1 vs. p=0.375 for partition coefficient). When comparing partition coefficient to T1 values individually, partition coefficient had better precision than T1 for all vials, with only the “post-contrast blood” vial not showing a significant difference (p=.096 for “post-contrast blood” vial vs. p<0.05 for all others). Tighter limits of agreement for partition coefficient over MOLLI T1 for both 1.5T and 3T via Bland-Altman analysis further demonstrated better reproducibility of partition coefficient over T1 values. Thus, relative measures, such as partition coefficient, displayed its superiority to direct T1 quantification for reproducibility. This will translate directly to better power and smaller sample size requirement for ECV over T1 in multi-center studies [20].

### Limitations

Several limitations in this study existed. First, no *in vivo* human data was used for the study. As the purpose of this study was to validate the accuracy and reproducibility of the acquisition technique itself, phantoms provided the most reproducible tools to examine and optimize the various parameters. To test the accuracy and precision across multiple scanners *in-vivo*, serial contrast-enhanced CMR studies need to be performed on the same subjects within a short time interval on different scanners. Such testing is unlikely due to the risks associated with multiple injections of MR contrast agents within a short time frame. Measurement errors are expected to be higher in patients compared with phantoms because of increased errors associated with physiological variations *in-vivo*. Additionally, ECV, rather than partition coefficient, is appearing to be more useful in the detection of subtle abnormalities present in diffuse myocardial fibrosis due to the large variability in patient hematocrit data [17]. As the only difference between partition coefficient and ECV is the hematocrit factor of roughly 0.4, the results in this study are easily translatable. Furthermore, only one time point post-contrast was chosen exemplarily. This coincides with most clinical protocols. Lastly, it is well known that T1 values could be affected by many factors, such as, room temperature [35]. Relative, and not absolute, accuracy and reproducibility measurements were reported in this study. Accuracy measures were standardized with IR-SE T1 and precision measures compared two MOLLIs with one another under same technical condition. This approach negates the potential influence of temperature and many other factors that might affect the results of comparison across different scanners.

## Conclusion

Our data indicates that significant accuracy and precision/reproducibility variation of T1 indices exists across scanners. This finding is consistent with across scanners variation of other quantitative CMR indices, such as apparent diffusion coefficient [36], fractional anisotropy and mean diffusivity [37], and voxel based morphometry [38]. MOLLI precision is preserved, and increases when partition coefficient is calculated. Over the entire range of T1 values expected to be encountered before and after gadolinium administration, the precision of partition coefficient, a relative T1 mapping index, was approximately 25% better than absolute T1 values. As multi-center studies using different MR platforms are ultimately required to assess the value of T1 measurements as a surrogate biomarker, pooling data together without quantification and control of the inter-site variability might affect statistical analysis which could require a larger sample size to compensate for such variability. In addition, serial assessment should be performed on the same platform. A pilot phantom study might be helpful to identify any scanners that may deviate from others with respect to T1 measurement.

## Competing interests

The authors declare they have no competing interests.

## Authors’ contributions

FR: data acquisition, data analysis, data interpretation, manuscript drafting; NK: study design, data acquisition, data interpretation, manuscript revision; NG: data interpretation, manuscript revision; MF: phantom design, manuscript revision; JH: data interpretation, manuscript revision; PK: data interpretation, manuscript revision; CYL: data interpretation, manuscript revision; CS: data interpretation, manuscript revision; JL: data interpretation, manuscript revision; DB: study design, data interpretation, manuscript revision; SL: study design, data acquisition, data analysis, data interpretation, manuscript revision. All authors read and approved the final manuscript.

## References

[B1] Ugander M Oki AJ Hsu LY Kellman P Greiser A Aletras AH Sibley CT Chen MY Bandettini WP Arai AE Extracellular volume imaging by magnetic resonance imaging provides insights into overt and sub-clinical myocardial pathology Eur Heart J 2012 33 10 1268 1278 10.1093/eurheartj/ehr481 22279111 PMC3350985

[B2] Arheden H Saeed M Higgins CB Gao DW Bremerich J Wyttenbach R Dae MW Wendland MF Measurement of the distribution volume of gadopentetate dimeglumine at echo-planar MR imaging to quantify myocardial infarction: comparison with 99mTc-DTPA autoradiography in rats Radiology 1999 211 3 698 708 10352594 10.1148/radiology.211.3.r99jn41698

[B3] Jerosch-Herold M Kwong RY Magnetic resonance imaging in the assessment of ventricular remodeling and viability Curr Heart Fail Rep 2008 5 1 5 10 10.1007/s11897-008-0002-4 18460288 PMC3955037

[B4] Sparrow P Messroghli DR Reid S Ridgway JP Bainbridge G Sivananthan MU Myocardial T1 mapping for detection of left ventricular myocardial fibrosis in chronic aortic regurgitation: pilot study AJR Am J Roentgenol 2006 187 6 W630 635 10.2214/AJR.05.1264 17114517

[B5] Iles L Pfluger H Phrommintikul A Cherayath J Aksit P Gupta SN Kaye DM Taylor AJ Evaluation of diffuse myocardial fibrosis in heart failure with cardiac magnetic resonance contrast-enhanced T1 mapping J Am Coll Cardiol 2008 52 19 1574 1580 10.1016/j.jacc.2008.06.049 19007595

[B6] Flett AS Hayward MP Ashworth MT Hansen MS Taylor AM Elliott PM McGregor C Moon JC Equilibrium contrast cardiovascular magnetic resonance for the measurement of diffuse myocardial fibrosis. Preliminary Validation in Humans Circulation 2010 122 2 138 144 10.1161/CIRCULATIONAHA.109.930636 20585010

[B7] Broberg CS Chugh S Conklin C Sahn DJ Jerosch-Herold M Quantification of diffuse myocardial fibrosis and its association with myocardial dysfunction in congenital heart disease Circ Cardiovasc Imaging 2010 3 6 727 734 10.1161/CIRCIMAGING.108.842096 20855860 PMC3048790

[B8] Lee JJ Liu S Nacif MS Ugander M Han J Kawel N Sibley CT Kellman P Arai AE Bluemke DA Myocardial T1 and extracellular volume fraction mapping at 3 tesla J Cardiovasc Magn Reson 2011 13 75 10.1186/1532-429X-13-75 22123333 PMC3269374

[B9] Schelbert EB Testa SM Meier CG Ceyrolles WJ Levenson JE Blair AJ Kellman P Jones BL Ludwig DR Schwartzman D Myocardial extravascular extracellular volume fraction measurement by gadolinium cardiovascular magnetic resonance in humans: slow infusion versus bolus J Cardiovasc Magn Reson 2011 13 1 16 10.1186/1532-429X-13-16 21375743 PMC3059279

[B10] Kawel N Nacif M Zavodni A Jones J Liu S Sibley CT Bluemke DA T1 Mapping of the myocardium: intra-individual assessment of the effect of field strength, cardiac cycle and variation by myocardial region J Cardiovasc Magn Reson 2012 14 1 27 10.1186/1532-429X-14-27 22548832 PMC3424109

[B11] Kawel N Nacif M Zavodni A Jones J Liu S Sibley CT Bluemke DA T1 Mapping of the myocardium: intra-individual assessment of post-contrast T1 time evolution and extracellular volume fraction at 3T for Gd-DTPA and Gd-BOPTA J Cardiovasc Magn Reson 2012 14 1 26 10.1186/1532-429X-14-26 22540153 PMC3405486

[B12] Sado DM Flett AS Banypersad SM White SK Maestrini V Quarta G Lachmann RH Murphy E Mehta A Hughes DA Cardiovascular magnetic resonance measurement of myocardial extracellular volume in health and disease Heart 2012 98 19 1436 1441 10.1136/heartjnl-2012-302346 22936681

[B13] Fontana M White SK Banypersad SM Sado DM Maestrini V Flett AS Piechnik SK Neubauer S Roberts N Moon JC Comparison of T1 mapping techniques for ECV quantification. Histological validation and reproducibility of ShMOLLI versus multibreath-hold T1 quantification equilibrium contrast CMR J Cardiovasc Magn Reson 2012 14 1 88 10.1186/1532-429X-14-88 23272651 PMC3552758

[B14] Nacif MS Turkbey EB Gai N Nazarian S van der Geest RJ Noureldin RA Sibley CT Ugander M Liu S Arai AE Myocardial T1 mapping with MRI: comparison of look-locker and MOLLI sequences J Magn Reson Imaging 2011 34 6 1367 1373 10.1002/jmri.22753 21954119 PMC3221792

[B15] Tofts PS Shuter B Pope JM Ni-DTPA doped agarose gel–a phantom material for Gd-DTPA enhancement measurements Magnetic resonance imaging 1993 11 1 125 133 10.1016/0730-725X(93)90420-I 8423715

[B16] Stanisz GJ Odrobina EE Pun J Escaravage M Graham SJ Bronskill MJ Henkelman RM T1, T2 Relaxation and magnetization transfer in tissue at 3T Magn Reson Med 2005 54 3 507 512 10.1002/mrm.20605 16086319

[B17] Kellman P Wilson JR Xue H Ugander M Arai AE Extracellular volume fraction mapping in the myocardium, part 1: evaluation of an automated method J Cardiovasc Magn Reson 2012 14 63 10.1186/1532-429X-14-63 22963517 PMC3441905

[B18] Schar M Kozerke S Fischer SE Boesiger P Cardiac SSFP imaging at 3 tesla Magn Reson Med 2004 51 4 799 806 10.1002/mrm.20024 15065254

[B19] Messroghli DR Plein S Higgins DM Walters K Jones TR Ridgway JP Sivananthan MU Human myocardium: single-breath-hold MR T1 mapping with high spatial resolution–reproducibility study Radiology 2006 238 3 1004 1012 10.1148/radiol.2382041903 16424239

[B20] Liu S Han J Nacif MS Jones J Kawel N Kellman P Sibley CT Bluemke DA Diffuse myocardial fibrosis evaluation using cardiac magnetic resonance T1 mapping: sample size considerations for clinical trials J Cardiovasc Magn Reson 2012 14 90 10.1186/1532-429X-14-90 23272704 PMC3552738

[B21] Messroghli DR Rudolph A Abdel-Aty H Wassmuth R Kuhne T Dietz R Schulz-Menger J An open-source software tool for the generation of relaxation time maps in magnetic resonance imaging BMC Med Imaging 2010 10 1 16 10.1186/1471-2342-10-16 20673350 PMC2919441

[B22] White SK Sado DM Flett AS Moon JC Characterising the myocardial interstitial space: the clinical relevance of non-invasive imaging Heart 2012 98 10 773 779 10.1136/heartjnl-2011-301515 22422587

[B23] Judd RM Kim RJ Extracellular space measurements with CMR imaging JACC Cardiovasc Imaging 2012 5 9 908 910 10.1016/j.jcmg.2012.04.007 22974803

[B24] Plein S Kidambi A Understanding LV remodeling following myocardial infarction: Are T1 maps by CMR the New guide? JACC Cardiovasc Imaging 2012 5 9 894 896 10.1016/j.jcmg.2012.07.006 22974801

[B25] Liu CY Liu YC Wu C Armstrong A Volpe GJ van der Geest R Liu Y Hundley WG Gomes AS Liu S Nacif MS Bluemke DA Lima JAC Evaluation of age related interstitial myocardial fibrosis with cardiac magnetic resonance contrast-enhanced T1 Mapping in the Multi-ethnic Study of Atherosclerosis (MESA) J Am Coll Cardiol 2013 10.1016/j.jacc.2013.05.078 PMC3807823 23871886

[B26] Wong TC Piehler KM Kang IA Kadakkal A Kellman P Schwartzman DS Mulukutla SR Simon MA Shroff SG Kuller LH Myocardial extracellular volume fraction quantified by cardiovascular magnetic resonance is increased in diabetes and associated with mortality and incident heart failure admission Eur Heart J 2013 [Epub ahead of print] 10.1093/eurheartj/eht193 PMC3945798 23756336

[B27] Karamitsos TD Piechnik SK Banypersad SM Fontana M Ntusi NB Ferreira VM Whelan CJ Myerson SG Robson MD Hawkins PN Noncontrast T1 mapping for the diagnosis of cardiac amyloidosis JACC Cardiovasc Imaging 2013 6 4 488 497 10.1016/j.jcmg.2012.11.013 23498672

[B28] Salerno M Janardhanan R Jiji RS Brooks J Adenaw N Mehta B Yang Y Antkowiak P Kramer CM Epstein FH Comparison of methods for determining the partition coefficient of gadolinium in the myocardium using T(1) mapping J Magn Reson Imaging 2012 38 1 217 224 23197434 10.1002/jmri.23875 PMC3899916

[B29] Piechnik SK Ferreira VM Dall’Armellina E Cochlin LE Greiser A Neubauer S Robson MD Shortened modified look-locker inversion recovery (ShMOLLI) for clinical myocardial T1-mapping at 1.5 And 3 T within a 9 heartbeat breathhold J Cardiovasc Magn Reson 2010 12 69 10.1186/1532-429X-12-69 21092095 PMC3001433

[B30] Flewitt JA Chow K Pagano JJ Green JD Friedrich MG Thompson RB Effect of systematic T1 errors on lambda caclulations: comparison of different T1 mapping techniques ISMRM 20th Annual Meeting 2012

[B31] Fitts M Breton E Kholmovski EG Dosdall DJ Vijayakumar S Hong KP Ranjan R Marrouche NF Axel L Kim D Arrhythmia insensitive rapid cardiac T(1) mapping pulse sequence Magn Reson Med 2012 [Epub ahead of print]. 10.1002/mrm.24586 23280998

[B32] Messroghli DR Greiser A Frohlich M Dietz R Schulz-Menger J Optimization and validation of a fully-integrated pulse sequence for modified look-locker inversion-recovery (MOLLI) T1 mapping of the heart J Magn Reson Imaging 2007 26 4 1081 1086 10.1002/jmri.21119 17896383

[B33] Wong TC Piehler K Meier CG Testa SM Klock AM Aneizi AA Shakesprere J Kellman P Shroff SG Schwartzman DS Association between extracellular matrix expansion quantified by cardiovascular magnetic resonance and short-term mortality Circulation 2012 126 10 1206 1216 10.1161/CIRCULATIONAHA.111.089409 22851543 PMC3464491

[B34] Kellman P Herzka DA Hansen MS Adiabatic inversion pulses for myocardial T1 mapping Magn Reson Med 2013 [Epub ahead of print] 10.1002/mrm.24793 PMC3775900 23722695

[B35] Bottomley PA Foster TH Argersinger RE Pfeifer LM A review of normal tissue hydrogen NMR relaxation times and relaxation mechanisms from 1–100 MHz: dependence on tissue type, NMR frequency, temperature, species, excision, and age Med Phys 1984 11 4 425 448 10.1118/1.595535 6482839

[B36] Sasaki M Yamada K Watanabe Y Matsui M Ida M Fujiwara S Shibata E Acute Stroke Imaging Standardization Group-Japan I Variability in absolute apparent diffusion coefficient values across different platforms may be substantial: a multivendor, multi-institutional comparison study Radiology 2008 249 2 624 630 10.1148/radiol.2492071681 18936317

[B37] Takao H Hayashi N Ohtomo K Effect of scanner in asymmetry studies using diffusion tensor imaging NeuroImage 2011 54 2 1053 1062 10.1016/j.neuroimage.2010.09.023 20850553

[B38] Focke NK Helms G Kaspar S Diederich C Toth V Dechent P Mohr A Paulus W Multi-site voxel-based morphometry–not quite there yet NeuroImage 2011 56 3 1164 1170 10.1016/j.neuroimage.2011.02.029 21324367

